# Cloning of Insertion Site Flanking Sequence and Construction of Transfer DNA Insert Mutant Library in *Stylosanthes Colletotrichum*

**DOI:** 10.1371/journal.pone.0111172

**Published:** 2014-10-31

**Authors:** Helong Chen, Caiping Hu, Kexian Yi, Guixiu Huang, Jianming Gao, Shiqing Zhang, Jinlong Zheng, Qiaolian Liu, Jingen Xi

**Affiliations:** 1 Institute of Tropical Bioscience and Biotechnology, Key Laboratory of Tropical Crop Biotechnology, Ministry of Agriculture, Chinese Academy of Tropical Agricultural Sciences, Haikou, China; 2 Environment and Plant Protection Institute, Key Laboratory of Integrated Pest Management on Tropical Crops, Ministry of Agriculture, Chinese Academy of Tropical Agricultural Sciences, Haikou, China; University of Kentucky College of Medicine, United States of America

## Abstract

*Stylosanthes* sp. is the most important forage legume in tropical areas worldwide. *Stylosanthes* anthracnose, which is mainly caused by *Colletotrichum gloeosporioides*, is a globally severe disease in stylo production. Little progress has been made in anthracnose molecular pathogenesis research. In this study, *Agrobacterium tumefaciens*-mediated transformation was used to transform *Stylosanthes colletotrichum* strain CH008. The major factors of the genetic transformation system of *S. colletotrichum* were optimized as follows: *A. tumefaciens*’ AGL-1 concentration (OD_600_), 0.8; concentration of *Colletotrichum* conidium, 1×10^6^ conidia/mL; acetosyringone concentration, 100 mmol/L; induction time, 6 h; co-culture temperature, 25°C; and co-culture time, 3 d. Thus, the transformation efficiency was increased to 300–400 transformants per 106 conidia. Based on the optimized system, a mutant library containing 4616 mutants was constructed, from which some mutants were randomly selected for analysis. Results show that the mutants were single copies that could be stably inherited. The growth rate, spore amount, spore germination rate, and appressorium formation rate in some mutants were significantly different from those in the wild-type strain. We then selected the most appropriate method for the preliminary screening and re-screening of each mutant’s pathogenic defects. We selected 1230 transformants, and obtained 23 strains with pathogenic defects, namely, 18 strains with reduced pathogenicity and five strains with lost pathogenicity. Thermal asymmetric interlaced PCR was used to identify the transfer DNA (T-DNA) integration site in the mutant that was coded 2430, and a sequence of 476 bp was obtained. The flanking sequence of T-DNA was compared with the *Colletotrichum* genome by BLAST, and a sequence of 401 bp was found in Contig464 of the *Colletotrichum* genome. By predicting the function of the flanking sequence, we discovered that T-DNA insertion in the promoter region of the putative gene had 79% homology with the aspartate aminotransferase gene in *Magnaporthe oryzae* (XP_003719674.1).

## Introduction

*Stylosanthes guianensis*, a diverse tropical and subtropical forage legume, is native to South America, Central America, and Africa. It is used for grazing cattle and raising livestock. Species of *Stylosanthes* are used for soil improvement through nitrogen fixation, reclaiming degraded wastelands, and water and soil conservation [1]–[3].

Introduction of *Stylosanthes* sp. to China from Australia, Africa, and South America began in the late 1960s and has continued to the present. *Stylosanthes* sp. is principally grown in Hainan and Guangdong Provinces as an annual crop for cut-and-carry forage, leaf meal, and hay [4].

Anthracnose of *Stylosanthes*, mainly caused by *Colletotrichum gloeosporioides* (Penz.) Penz. & Sacc., has been the most significant biotic factor limiting the production, persistence, and utilization of *Stylosanthes* in several countries [5]. The fungus initially infects leaves via an appressorium that develops from the germinating spore on the plant surface, followed by turgor-driven penetration of the cuticle. Fungal colonization on the leaf tissue follows and is associated with host cell necrosis, leading to a blight-like symptom and the formation of spore masses as acervuli [6], [7]. Research in Australia, Colombia, Brazil, and China has identified two biotypes of *C. gloeosporioides* infecting *Stylosanthes* sp. [8]–[13]. Similarly, biotypes A and B and putative biotype C from Africa have been described [14]. The diversity among strains pathogenic on *Stylosanthes* and their relationship with other strains were analyzed at the molecular level using various markers, such as dsRNA [15], RFLP [16], [17], RAPD [14], [18]–[20], and ITS [21]. The diversity among the pathogen population from Brazil, Colombia, China, and India is extensive [19]. *Agrobacterium tumefaciens*-mediated transformation (ATMT) has been used to identify mutants of *C. gloeosporioides* impaired in pathogenicity to gain more insight into the molecular mechanisms of *C. gloeosporioides* pathogenesis [22].

ATMT is a suitable and efficient technique for insertion mutagenesis, genetic mapping, and related research in filamentous fungi [23]. ATMT has been used to transform over 50 different fungal species since it was first reported [24]. The advantages of ATMT are as follows: first, *A. tumefaciens* directly transforms fungal spores, hyphae, or tissues without protoplast preparation; second, the integration of transfer DNA (T-DNA) into the chromosome is random and generally involves a single copy, which can easily isolate and identify the insertion locus; third, ATMT is competent for the transformation of high-molecular-weight exogenous DNA [25], [26]. *Agrobacterium*-mediated T-DNA tagging has been developed as a powerful tool for both random and targeted gene disruption; it is increasingly being regarded as the system of choice for many fungi [24]. *Agrobacterium*-mediated T-DNA tagging is a high-throughput system for identifying and analyzing novel genes [27]–[29], and the key for its success is the discovery of T-DNA-inserted mutants with altered phenotypes.

Traditional control measures for *Stylosanthes* anthracnose mainly involve chemical prevention and agricultural measure control. The use of pesticide produces the most direct effect, but it may easily cause a series of problems, such as pollution of the ecological environment. Theoretically, the most cost-effective and efficient control method is the cultivation of disease-resistant *S. guianensis* varieties. However, this method requires intensive manpower, material resources, and time. A single disease-resistant variety of *S. guianensis* cannot overcome the diversity and variability of *Stylosanthes Colletotrichum*. The resistance of cultivated disease-resistant varieties can only keep about 5a because *Colletotrichum* suffers from variation easily. Therefore, exploration on pathogenesis and causes for variation in *Stylosanthes colletotrichum* at the levels of molecular biology and functional genomics is of scientific significance and application value to effectively cultivate new varieties of *S. guianensis* with long-term disease resistance, and formulate permanent strategies for the reasonable control of *Stylosanthes* anthracnose.

Based on the constructed anthracnose genetic transformation system of *S. guianensis*, this study aimed to clone genes related to pathopoiesia of pathogenic bacteria. In this study, we utilized and inserted *A. tumefaciens*-mediated T-DNA, which contains an anti-chlorimuronethyl gene, into genes of *Stylosanthes colletotrichum gloeosporiodes* Penz strain CH008 with strong pathogenicity to generate insertion mutations, construct a library of mutants from *Stylosanthes colletotrichum gloeosporiodes* Penz strain CH008, and provide many mutation materials for future studies on functional genes analysis. Based on the selection of mutants related to pathogenicity in the mutant library, PCR and Southern blot were used for molecular verification. Flanking sequences of T-DNA insertion in virulence genes were obtained via thermal asymmetric interlaced PCR (TAIL-PCR). However, sequencing work on the whole genome sequence of *S. colletotrichum* remains unfinished. To predict information such as related gene functions of pathopoiesia, we compared the flanking sequences with the *Colletotrichum* gene libraries of known sequences, and used BLAST to analyze the homologous sequences. The results of this study could provide a basis for further investigations on functions of disrupted genes.

## Materials and Methods

No specific permission was required for the sampling locations of this study. Moreover, ethical approval for this study was not required because we did not handle or collect animals involved in any animal welfare regulations, and no endangered or protected species were used as samples in the experiments. The study was conducted in Institute of Tropical Bioscience and Biotechnology, Chinese Academy of Tropical Agricultural Sciences (CATAS), Haikou, Hainan Province.

### Materials

#### Bacterial strains and plasmid for tests

The bacterial strain used was *A. tumefaciens* AGL-1. The bacterium included a pSULF.gfp plasmid, which uses pCAMBIAl300 as a framework and contains the ILV1 gene of chlorimuronethyl resistance marker and binary carriers of reporter gene GFP. The strain was constructed by the Sainsbury Laboratory of John Innes Center in Norwich, Britain [30], and donated by Professor He Zhaozu of Hainan University. The recipient bacterium was collected from main flower and grass planting areas in China. C*olletotrichum* strain CH008 with strong pathogenicity was obtained via purification and single spore isolation [20].

#### Culture medium

PDA and LB were prepared by conventional methods. Details about the preparation of minimal medium (MM), induction medium (IM), and selective medium can be found in the literature [31], [32].

#### *S. guianensis* for tests

Highly sensitive *S. guianensis* variant AFT3309 was collected from the Forage Germplasm Garden of CATAS. The collected leaves were seven-day-old ternate compound leaves. Each compound leaf contained three small leaves. One small leaf was connected to a wild-type strain with strong pathogenicity, whereas the other two were connected to a transformant strain.

### Methods

#### Construction of T-DNA insertion mutant library of *S. colletotrichum*

ATMT for *S. colletotrichum* was performed according to the methods of Hu XW [33] and Lin CH [34].

**Determination of optimum working concentration of antibiotics (chlorimuronethyl).** DCM plates with various concentrations of chlorimuronethyl (0, 5, 10, 15, 20, 30, 40, 50, and 60 µg/mL) were prepared. Approximately 1 µL of activated spore suspension of *S. colletotrichum* strain CH008 (concentration, 10^6^ spores/mL) was dropped into the center of DCM containing various concentrations of chlorimuronethyl (each concentration gradient was investigated in triplicate), and cultivated at 28°C for 5 d to observe the growth of *C. gloeosporiodes* Penz and determine the working concentration.

**Effects of acceptor materials on transformation**. We used six different levels (0.2, 0.4, 0.6, 0.8, 1.0, and 1.2) for the OD_600_ value of *A. tumefaciens*, and four different levels (10^4^, 10^5^, 10^6^, and 10^7^ spores/mL) of *S. colletotrichum gloeosporiodes* spore fluid concentration. The effects of 24 different combinations on the transformation efficiency were examined. Experiments were performed in triplicate.

**Effects of *Agrobacterium* induction time on transformation.** The effects of seven different induction times of *Agrobacterium* (3, 5, 6, 7, 8, 10, and 12 h) on the transformation efficiency were determined. Experiments were performed in triplicate.

**Effects of acetosyringone (AS) concentrations on transformation.** The effects of four different levels of AS concentration (0, 100, 150, and 200 µmol/L) on the conversion efficiency were determined. Experiments were performed in triplicate.

**Effects of co-culture time on transformation.** The effects of seven different co-culture times (1, 2, 3, 4, 5, 6, and 7 d) on the conversion efficiency were determined. Experiments were performed in triplicate. Co-culture was conducted following the methods of Merer [35].

**Effects of co-culture temperature on transformation during co-culture.** The effects of five different temperatures (20°C, 22°C, 25°C, 26°C, and 28°C) on the conversion efficiency were determined. Experiments were performed in triplicate. Co-culture was performed according to the methods of Merer [35].

**Verification of transformants.**

**A. PCR**

Total DNA of *S. colletotrichum gloeosporiodes* Penz transformants was extracted according to the conventional hexadecyltrimethylammonium bromide (CTAB) method. According to the GFP gene sequence in the plasmid pSuLF·GFP, primer pairs were designed. The primers were as follows:

GFP-F: 5′-TACTGCAGATGGTGAGCAAGGGCGAG-3′
GFP-R: 5′-CGGGATCCCTTGTACAGCTCGTCCATG-3′

For PCR reaction, a 20 µL system was used. The following reaction conditions were used: pre-degeneration at 94°C for 3 min, degeneration at 94°C for 45 s, annealing at 58°C for 45 s, extension at 72°C for 1 min, and final extension at 72°C for 10 min after 30 cycles. Samples were stored at 10°C.

**B. Fluorescence microscopy test**

Thirty transformants from the mutant library were randomly selected and cultivated on PDA with illumination at 28°C. Sterile water (1 mL) was dropped on the bacterial colony, and a pipette was used to carefully blow and mix the colony. Subsequently, 3 µL of the bacterial colony was placed on a clean glass slide. The slide was covered with a cover slip, and examined by confocal fluorescence microscopy. An excitation wavelength of 400–500 nm was used.

### Analysis of T-DNA insertion mutant library of *S. colletotrichum*

#### Analysis of the copy number of T-DNA insertion mutants (Southern hybridization)

Mycelia of *Colletotrichum* mutants were inoculated to PDA/CM fluid culture medium containing 200 µg/mL cephalosporin, 50 µg/mL tetracycline, and 10 µg/mL chlorimuronethyl. The medium was shaken at 28°C and 150 rpm for 7 d. Filter paper was used for filtration, and mycelial pellets were collected. Total DNA of *S. colletotrichum gloeosporiodes* transformants was extracted according to the conventional CTAB method. A detail protocol of Southern blot was performed following the specifications of DIG-High Prime DNA Labeling and Detection Starter Kit I.

#### Analysis of the growth rate of mutants

Purified untransformed CH008 was cultivated, and 30 transformants were randomly selected and cultivated on PDA plates for 5 d. The concentration of spore fluid was adjusted to 10^4^ spores/mL. A pipette was used to extract 2 µL of spore fluid, which was inoculated onto a PDA plate. The plate was incubated at 28°C. After 4 d of cultivation, the colony diameter was measured once a day. The difference in colony diameter of two adjacent days was determined to represent the colony growth rate, and each strain was analyzed three times. The growth of colonies was observed for 9 d, and photos of their morphology were taken.

#### Sporulation quantity and spore morphology of mutants

Thirty transformants and untransformed CH008 were cultivated according to the methods specified above. After 6 d, sterile water was used to dilute spore fluid. A blood counting chamber was used for counting. Spore morphology was observed, and differences in spore morphology were recorded.

#### Conidial germination and appressorium formation of mutants

Fresh conidia suspension liquid was obtained using a water washing method. Its concentration was adjusted to 1.0×10^5^ and 1.0×10^6^ conidia/mL. Suspension liquid was dropped to a clean glass slide, blotted with a piece of bibulous filter paper, and incubated at 28°C. A total of 100 conidia were statistically analyzed, and the appressorium formation rate of germinated spores was recorded. Analyses were performed in triplicate. Samples were observed at 4, 6, 8, 10, and 12 h.

### Selection of pathogenicity-defective transformants of *S. colletotrichum* mutant library and analysis of flanking sequences at T-DNA insertion sites

#### Preliminary screening and re-screening of pathogenic defects of mutants

For preliminary screening, two inoculation methods, namely, spore fluid and mycelium cake, were used. In the spore fluid method, sterile water was used to wash *Colletotrichum* conidium cultivated on PDA for 5 d to prepare 5×10^5^ CFU/mL suspension liquid. Conidium liquid (1 µL) was dropped on *S. guianensis* leaves with punctured parts and normal ones. Mycelium cakes at the edge of *Colletotrichum* bacterial colony were cultivated on PDA for 5 d (diameter, 5 mm). The surface of a mycelium cake, which contained hyphae, was attached to *S. guianensis* leaves with punctured parts and normal ones to observe morbidity. This experiment was performed in triplicate. The re-screening method was similar to preliminary screening, except that it was performed using potted and complete plants. According to the comparison of different inoculation methods, the most appropriate one was selected and applied for preliminary screening and re-screening of the pathogenic defects of each mutant.

#### Cloning of flanking sequences at T-DNA insertion sites of mutant strains with pathogenic defects

TAIL-PCR was used for amplification of the flanking sequences at T-DNA insertion sites. Random primers, composition of nested primers at left and right boundaries, and PCR procedures were based on the methods of Mullins [36]. After the PCR products accepted a 1.0% AGE test, cloning and sequencing were conducted. BLAST comparison was implemented for the obtained sequence.

## Results

### Construction of T-DNA insertion mutant library of *S. colletotrichum*

#### Optimum concentration of chlorimuronethyl

**Figure 1:**
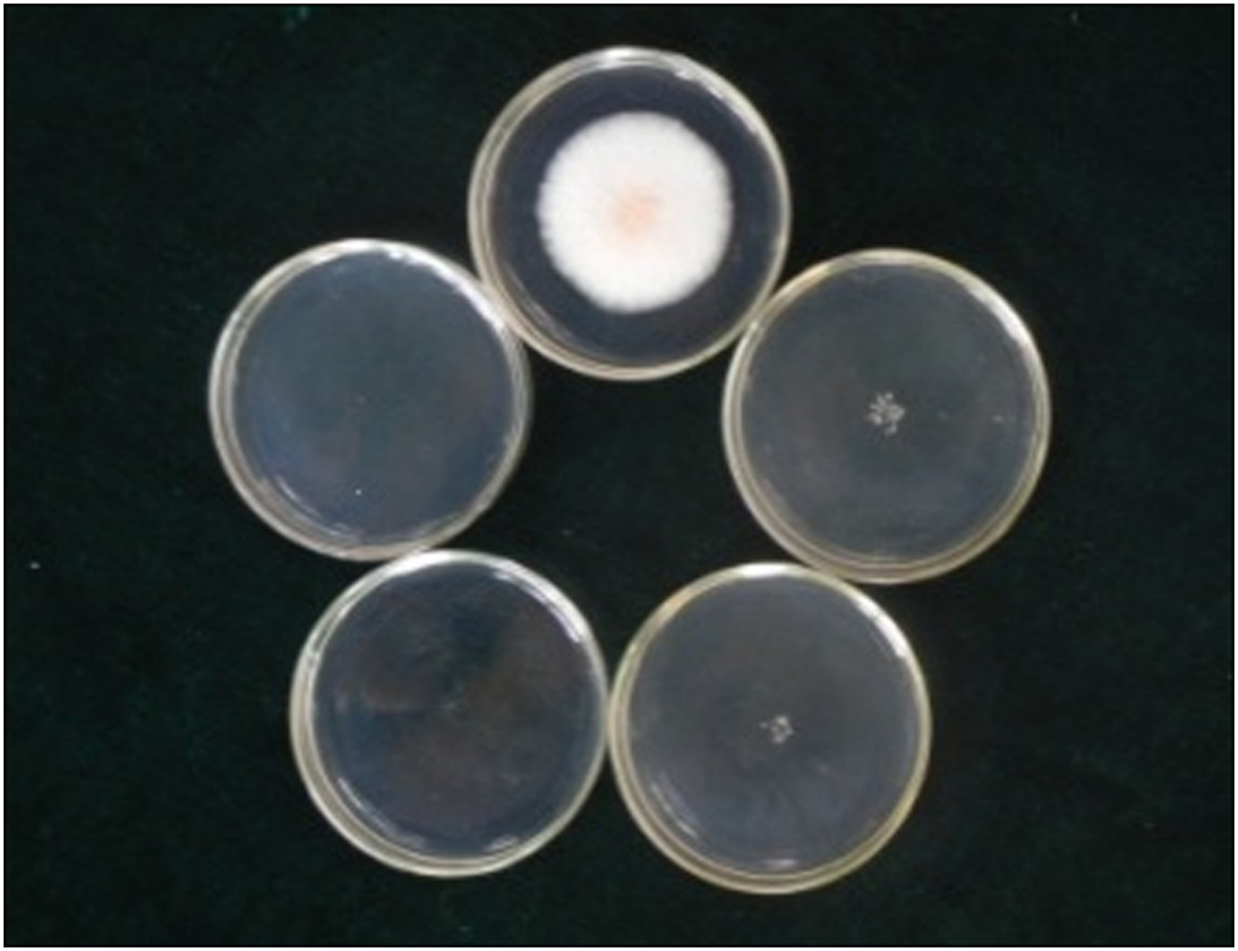
Tolerance test of a wild-type strain on medium containing concentration gradients of chlorimuronethyl. Note (from the top, clockwise): DCM plates (concentrations of 0, 5, 10, 20, and 30 µg/mL); cultivation was carried out at 28°C for 4 d.

In accordance with Figure 1, *S. colletotrichum* CH008 was inhibited at 5 µg/mL chlorimuronethyl and this strain nearly showed no growth at 10 µg/mL chlorimuronethyl. Thus, the final concentration selected by the culture medium was 10 (preliminary screening) and 20 µg/mL (re-screening).

#### Effects of acceptor materials on transformation

**Figure 2:**
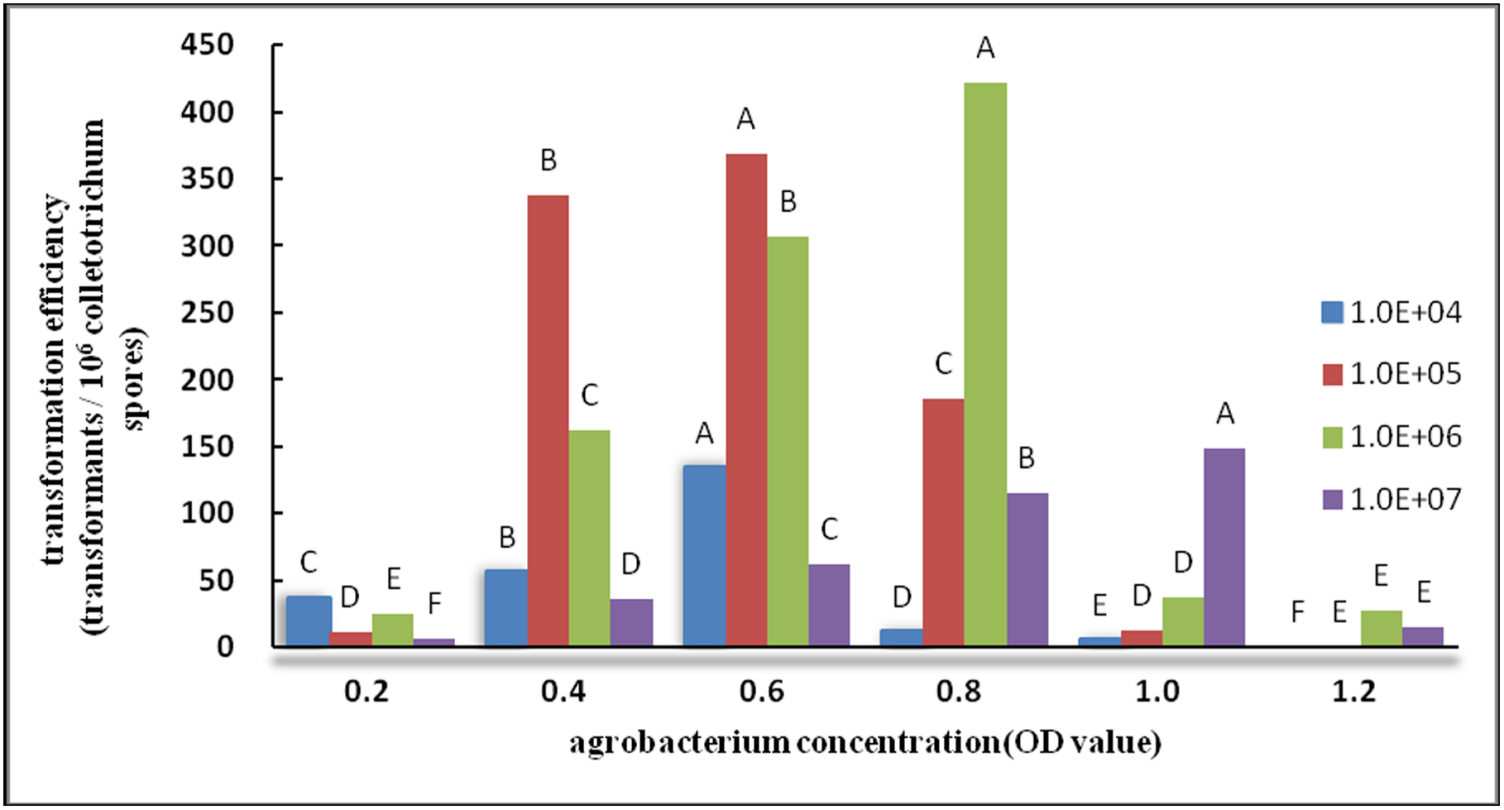
Effect of *Agrobacterium* concentration on transformation efficiency. Note: In the same series (four series), the same capital letters indicate no significant difference (p>0.01), whereas different capital letters indicate significant differences (p<0.01).

Results of the differences in concentrations of *A. tumefaciens* and *Colletotrichum* spore liquid were analyzed by ANOVA (similar to Duncan’s new multiple range method below). According to the related results, the difference in experimental results was significant at p<0.01. Based on Figure 2, each OD value had a corresponding and appropriate spore liquid concentration within an appropriate range of OD_600_ (0.4–0.8). A high concentration of spore liquid resulted in a high transformation efficiency. In particular, when *Agrobacterium* OD_600_ was equal to 0.8 and the number of *Colletotrichum* CH008 spores was 10^6^ spores/mL, the results were significantly higher than the other combinations, and the transformation efficiency peaked. This result may be related to the growth cycle of *Agrobacterium* and features of *Colletotrichum* CH008 spores. When the OD_600_ values of *Agrobacterium* were 1.0 and 1.2, the transformation efficiency showed a reducing trend with false-positive results.

#### Effects of induction time on transformation

**Figure 3:**
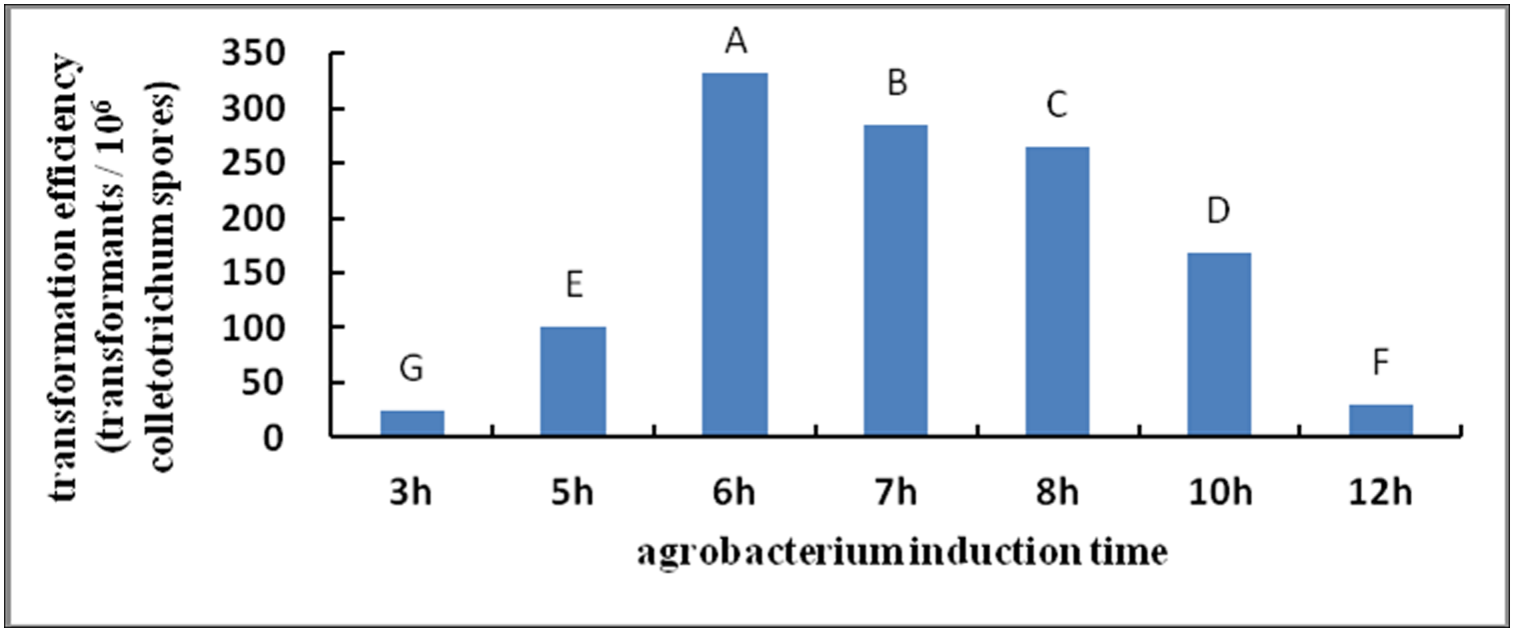
Effect of induction time on transformation efficiency. Note: Different capital letters indicate significant differences (p<0.01).

By inducing the activation and expression of genes at the Vir region of *Agrobacterium*, AS promoted T-DNA processing and transfer so that *Agrobacterium* T-DNA could enter the target genome and integrate with it more easily. An appropriate induction time was directly related to the ability of AS to activate the Vir region sufficiently, and affected the efficiency of final recombination. The effect was most significant in this experiment, and the transformation efficiency peaked at 6 h of induction and transformation (Figure 3). However, extending the induction time did not improve the transformation efficiency. This finding may be due to the fact that the culture time of *Agrobacterium* was too long, so *Agrobacterium* gradually entered a decline phase and affected the transformation efficiency.

#### Effects of AS on transformation

**Figure 4:**
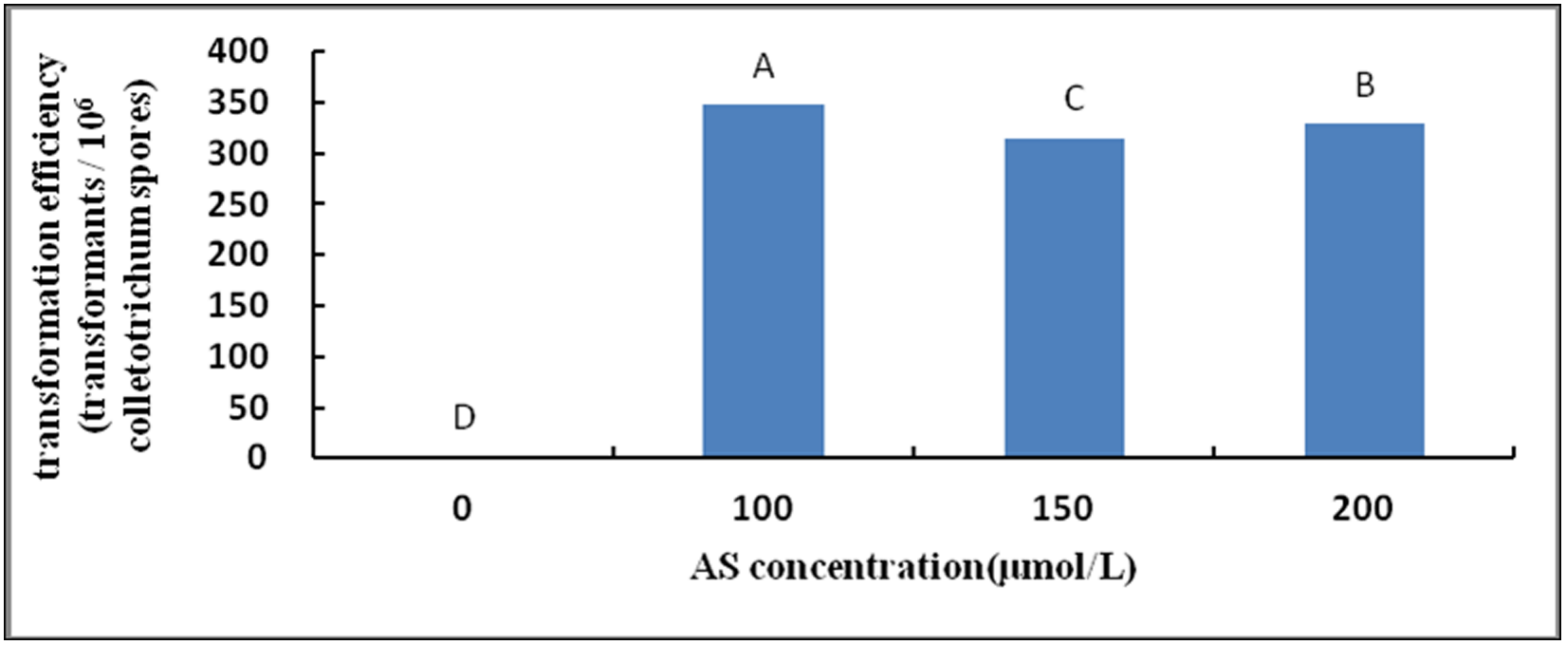
Effect of AS concentrations on transformation efficiency. Note: Different capital letters indicate significant differences (p<0.01).

When the AS concentration was 100 µmol/L (Figure 4), the results were significantly higher than those at other AS levels. However, the transformation efficiency did not improve as the concentration increased.

#### Effects of co-culture time on transformation

**Figure 5:**
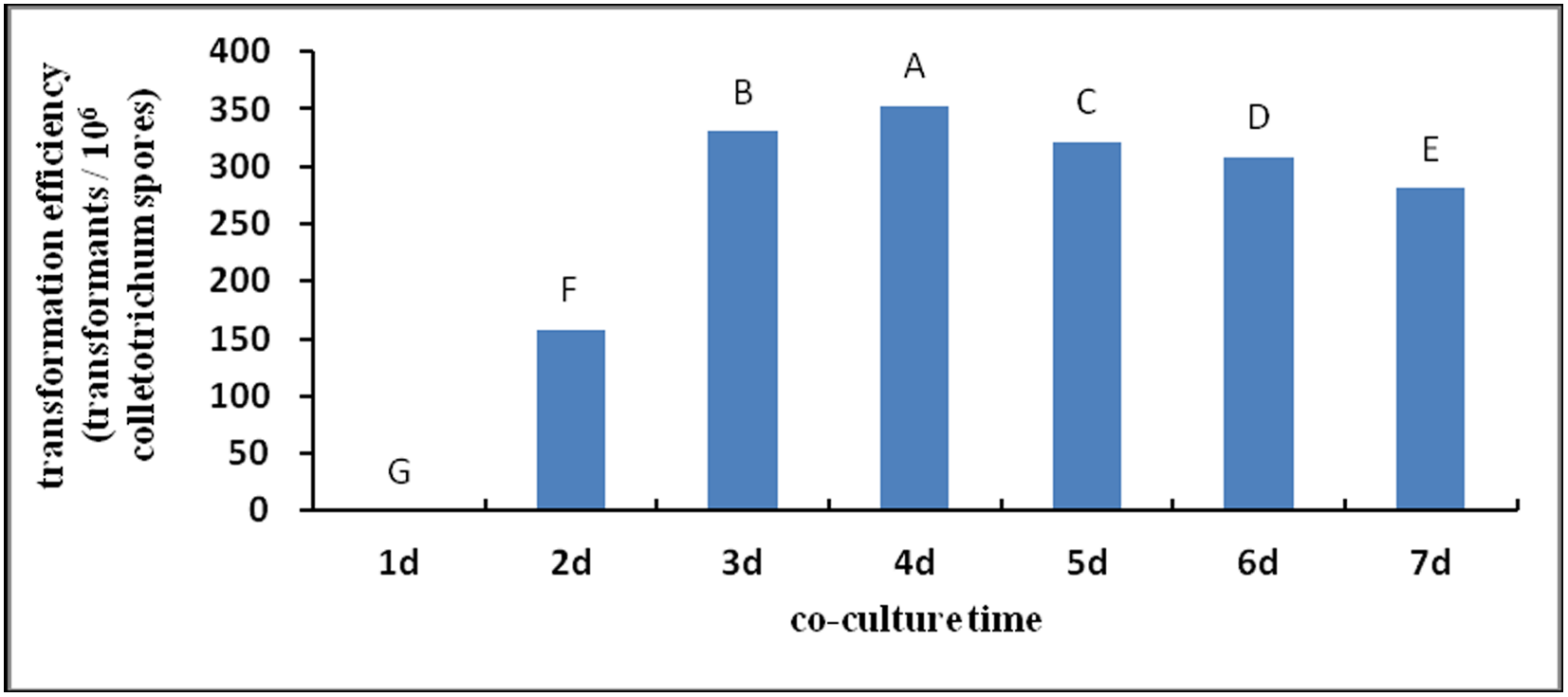
Effect of co-culture time on transformation efficiency. Note: Different capital letters indicate significant differences (p<0.01).

According to Figure 5, the transformation efficiency peaked when *S. colletotrichum* and *Agrobacterium* were co-cultured for 3–4 d, and the results were highly significant.

#### Effects of temperature on transformation during co-culture

**Figure 6:**
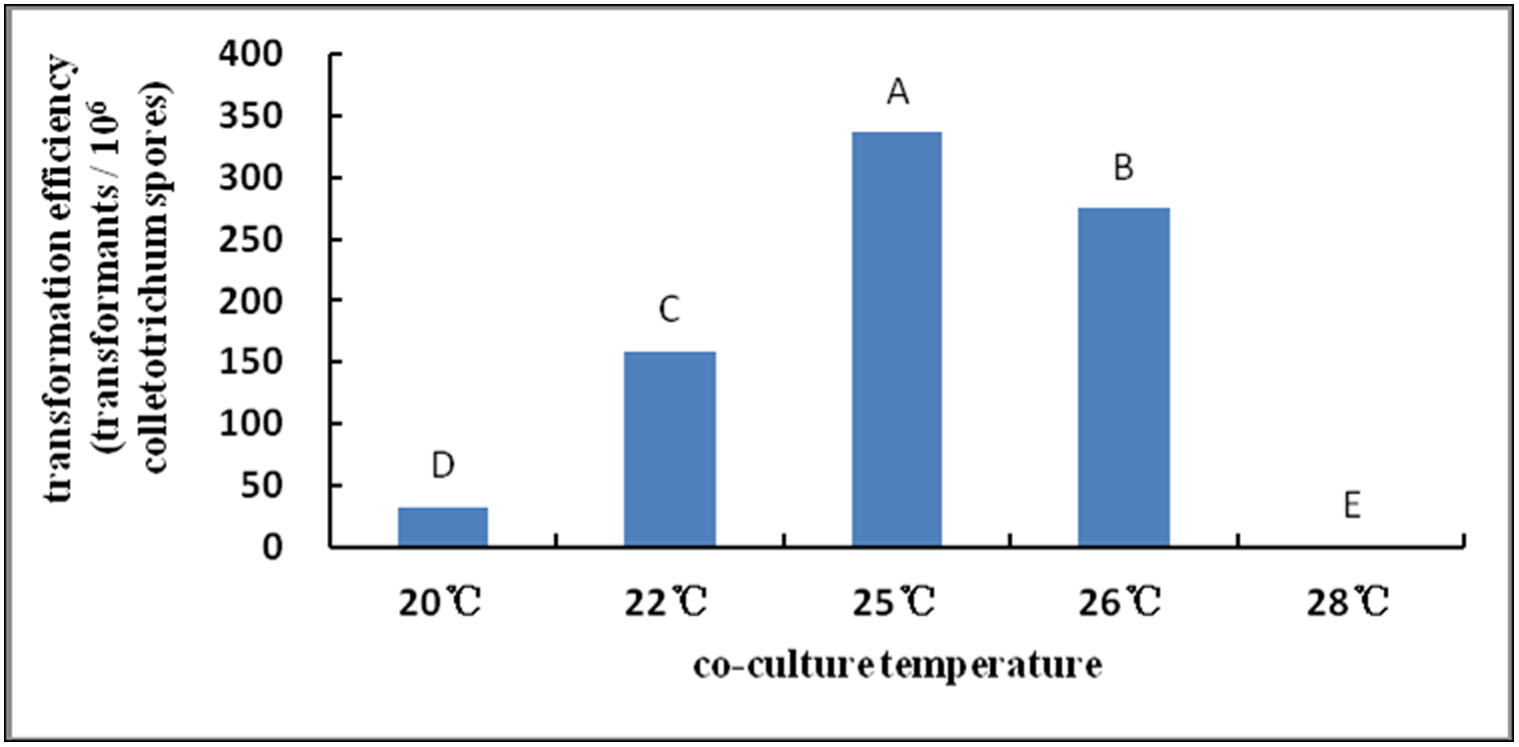
Effect of co-culture temperature on transformation efficiency. Note: Different capital letters indicate significant differences (p<0.01).

In this experiment (Figure 6), the optimum transformation efficiency was obtained at a co-culture temperature of 25°C. Its effect was most significant at this temperature.

#### PCR

**Figure 7:**
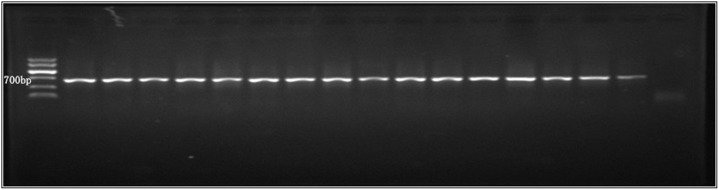
Verification of PCR results. Note: Marker v is on the left, followed by the positive control of plasmid and transformants, and untransformed CH 008 is on the right.

Among 20 selected converter strains, the positive control and all transformants demonstrated bright and clear strips at 750 bp. Thus, the T-DNA insertion rate was 100% (Figure 7).

#### Verification of fluorescence of transformant’s conidia

**Figure 8:**
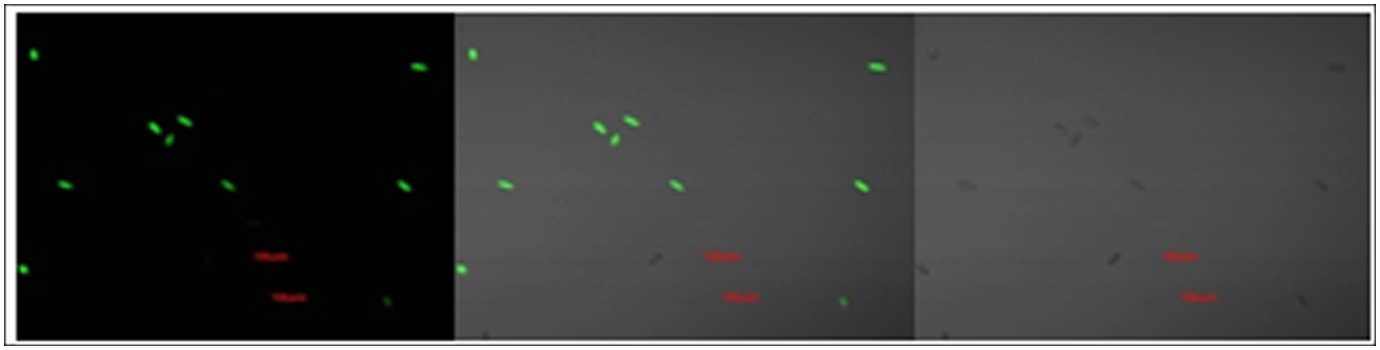
GFP fluorescence of *S. colletotrichum* T-DNA transformants.

Thirty transformants were randomly selected. After sporulation, conidia were examined under confocal fluorescence microscopy at an excitation wavelength of 400–500 nm (Figure 8). Spores of 13 transformants were bright under fluorescence, which indicates that GFP genes were carried to and integrated with *S. colletotrichum gloeosporiodes* Penz genome, and exhibited good expression.

This study optimized the genetic transformation system conditions of *S. colletotrichum*. The optimized conditions were as follows: AGL-1 concentration of *A. tumefaciens* (OD_600_), 0.8; concentration of *Colletotrichum* conidium, 1×10^6^ conidia/mL; AS concentration, 100 mmol/L; induction time, 6 h; co-culture temperature, 25°C; and co-culture time, 3 d. The T-DNA insertion mutant library of *S. colletotrichum* was constructed successfully using the genetic transformation system. The transformation efficiency was determined to be 300–400 transformants/10^6^ *Colletotrichum* spores.

### Analysis of *S. colletotrichum* T-DNA insertion mutant libraries

The genetic transformation system was used to obtain 4616 *S. colletotrichum* genetic transformants. Some of these transformants were then selected for analysis.

#### Analysis of the copy number of T-DNA insertion mutants

**Figure 9:**
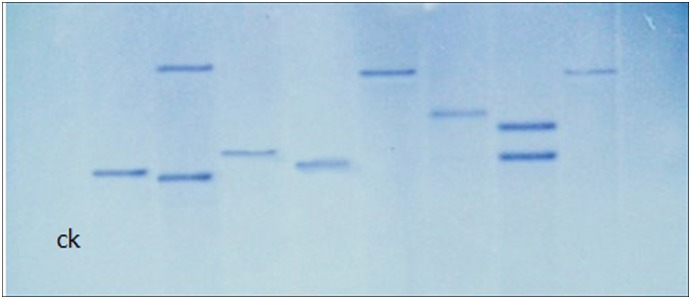
Southern blot analysis of genomic DNA from untransformed CH008 (CK) and transformed isolates.

The results of Southern blot are shown in Figure 9. Among eight randomly selected transformants, six demonstrated a single strip and the remaining two had two strips. Untransformed CH 008 and one transformant had no strip. These results indicate that most T-DNA insertions had a single locus, which could aid in amplification for sequences of insertion sites via Tail-PCR.

#### Genetic stability

**Figure 10:**
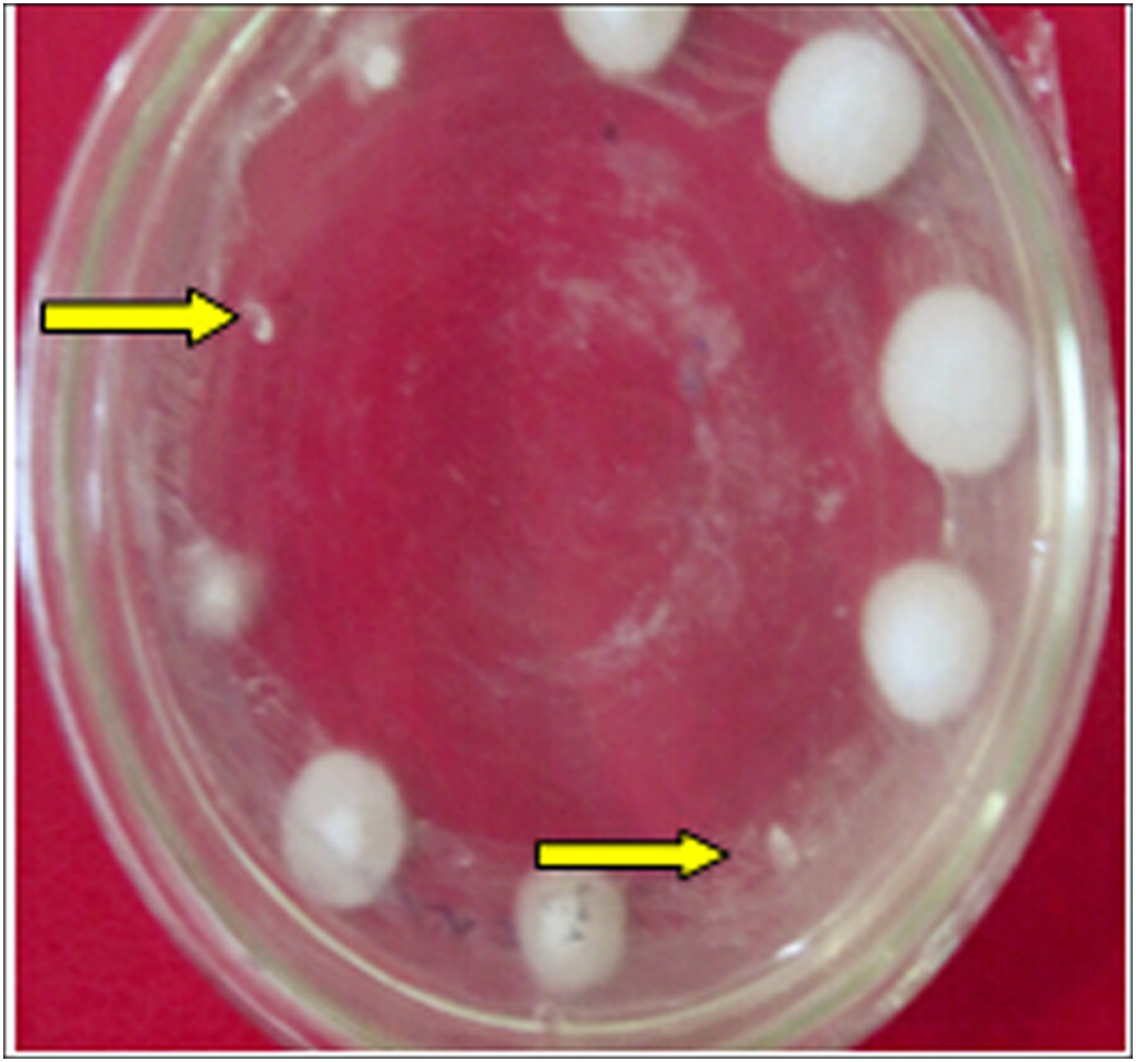
Determination of transformants’ genetic stability (arrows indicate wild strains).

After cultivating untransformed and mutant strains in PDA plates without chlorimuronethyl for 10 generations, they were inoculated to screening plates with chlorimuronethyl. The transformants grew normally (Figure 9), whereas the untransformed strains did not grow (indicated by the arrow in Figure 10). These findings show that resistance could be stably inherited in transformants.

#### Growth rate and colony morphology of transformants

Untransformed CH008 was cultivated, and 30 transformants were randomly selected. Thus, we observed an obvious difference in the growth rate of transformants. Specifically, mutant 1561 had the lowest growth rate, and its growth rate was much lower than that of untransformed CH008. Transformant 328 exhibited the highest growth rate (Table 1). Some strains demonstrated morphological variation, such as white hyphae that did not generate spores, greyish green hyphae, and yellow hyphae. However, the proportion of such strains was small.

**Table 1:** Comparison of the growth rate of untransformed strains and transformants.

Approaching growth rate of untransformed strains		High growth rate of untransformed strains		Low growth rate of untransformed strains	
Ch008	0.80 cm/d	888	1.06 cm/d	1561	0.37 cm/d
1130	0.76 cm/d	328	1.40 cm/d	3532	0.53 cm/d
993	0.83 cm/d	2715	1.13 cm/d	678	0.40cm/d
1477	0.80 cm/d	3590	1.20cm/d	3425	0.60 cm/d
3200	0.87 cm/d	3416	1.23 cm/d	1801	0.56 cm/d
2181	0.73 cm/d	1761	1.16 cm/d	3393	0.46 cm/d
3616	0.86 cm/d	3605	1.00 cm/d	604	0.43 cm/d

Note: Data in the table are average values of three measurements.

#### Sporulation quantity of mutants

The sporulation quantities of wild strains and mutants were determined, and the results are shown in Table 2. The sporulation quantity of transformants 2181, 2881, 2561, 844, and 888 was much higher than that of untransformed CH008, whereas the sporulation quantity of transformants 1477, 1561, and 2097 was lower than that of untransformed CH008. Transformants 3443 and 993 showed nearly no sporulation quantity.

**Table 2:** Comparison of transformants’ ability to produce spores.

Strain number	Ch008	2097	1447	1477	2181	961	2881	888	993
Spore concentration	1.05×10^6^	4.00×10^4^	2.15×10^5^	1.00×10^6^	2.15×10^7^	6.03×10^4^	1.67×10^7^	1.50×10^5^	0
Strain number	3590	3661	3605	2561	3443	844	1561	1761	604
Spore concentration	2.33×10^6^	2.34×10^5^	2.50×10^5^	3.45×10^7^	0	1.75×10^7^	2.35×10^5^	5.40×10^7^	1.70×10^5^

#### Conidial germination and appressorium formation in mutants

**Figure 11:**
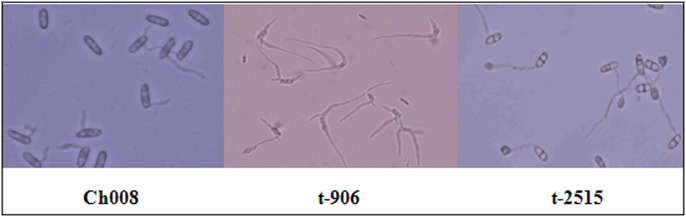
Conidial germination and appressorium formation after 8 h.

The results show that wild-type conidia and 10 randomly selected transformant spores could germinate after some time. The time that most of the transformants took to germinate was shorter than that of the wild-type strains, and the average length of germ tube growth in some transformants was longer than that of the wild-type strains. Three mutant strains demonstrated both low germination and appressorium formation rates, namely, t-960, t-604, and t-2327. By contrast, seven mutant spores (t-2393, t-2515, t-906, t-888, t-1130, t-2416, and t-3616) showed no significant difference from wild-type strains. In addition, wild-type strains and most transformants grew one to two germinal tubes from both ends of spores, and 80% of them demonstrated appressorium formation. Spore germination of mutant t-906 was abnormal; this mutant had three to four germinal tubes and its germination rate was high, but it germinated few appressoria (Table 3 and Figure 11).

**Table 3:** Comparison of conidial germination and germination rate.

Strain name	Initial germination time	Germination rate	Length and number of germinal tubes
Ch008	8 h	90–100	55–110 µm, 1
t-2515	6 h	80–90	110–300 µm, 2
t-2393	8 h	70–80	200–300 µm, 2
t-2327	6 h	40–50	300–400 µm, 1
t-888	6 h	70–80	200–250 µm, 2
t-906	4 h	95–100	300–400 µm, 3–4
t-960	8 h	60–70	30–45 µm, 1
t-1130	8 h	70–80	100–255 µm, 2
t-2416	6 h	90–100	110–350 µm, 2
t-3616	6 h	70–80	20–100 µm, 1
t-604	4 h	40–60	110–300 µm, 2

### Selection of transformants of the *S. colletotrichum* mutant library with pathogenic defects and analysis of flanking sequences at T-DNA insertion sites

#### Preliminary screening of mutants with pathogenic defects

**Figure 12:**
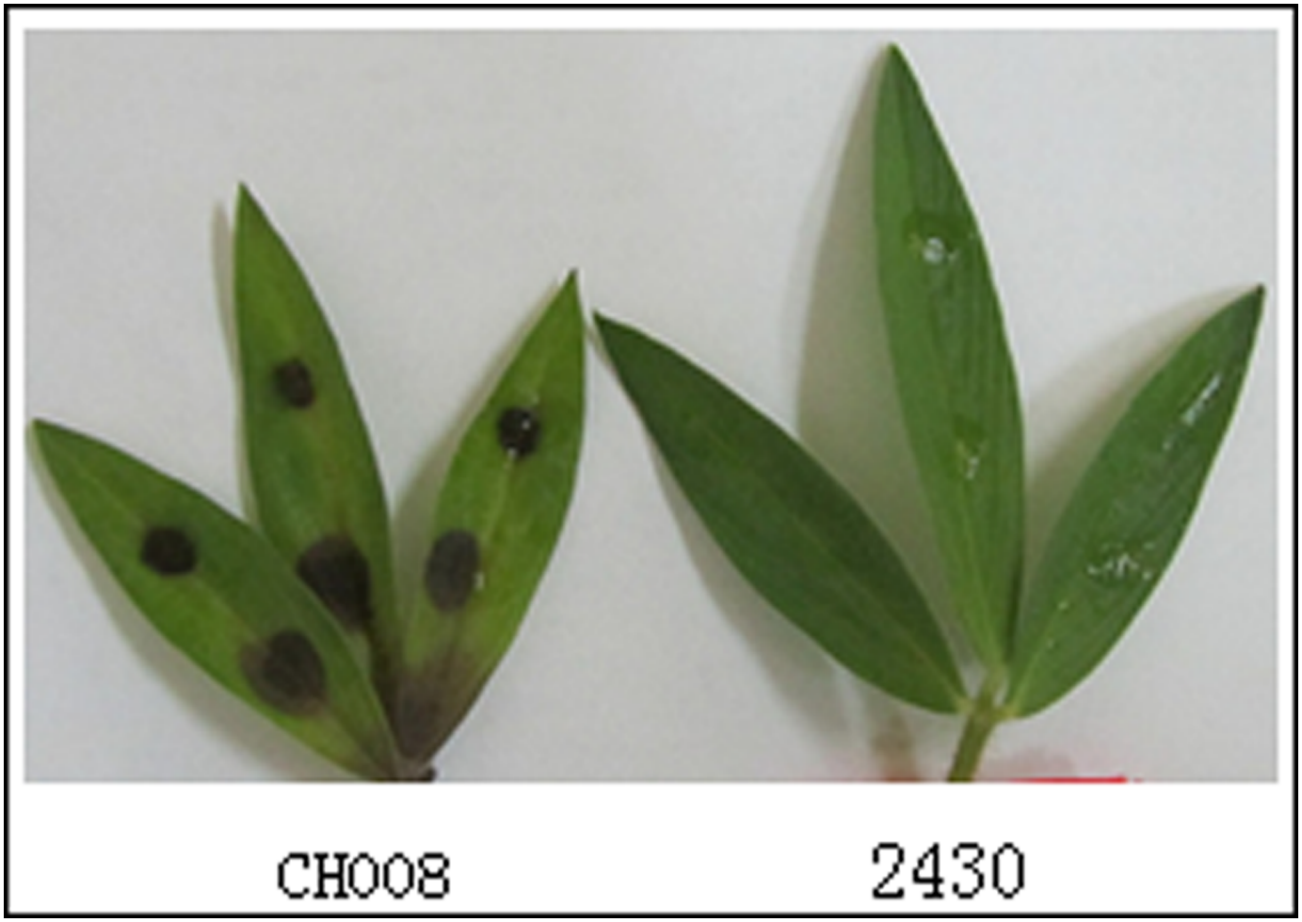
Determination of virulence during preliminary screening.

Comparison of inoculation methods showed that the most appropriate preliminary screening method was the use of the wild-type strain CH008 and transformant spore liquid to infect punctured parts of leaves of *S. guianensis*, and select mutants with lost pathogenicity. Using this method, we selected 1230 transformants and obtained 23 strains with pathogenic defects (18 strains with reduced pathogenicity and five strains with lost pathogenicity). Figure 12 shows that brown scabs formed at the infected part of wild strain CH008, whereas transformant 2430 lost its infection ability.

#### Re-screening of pathogenicity determination

**Figure 13:**
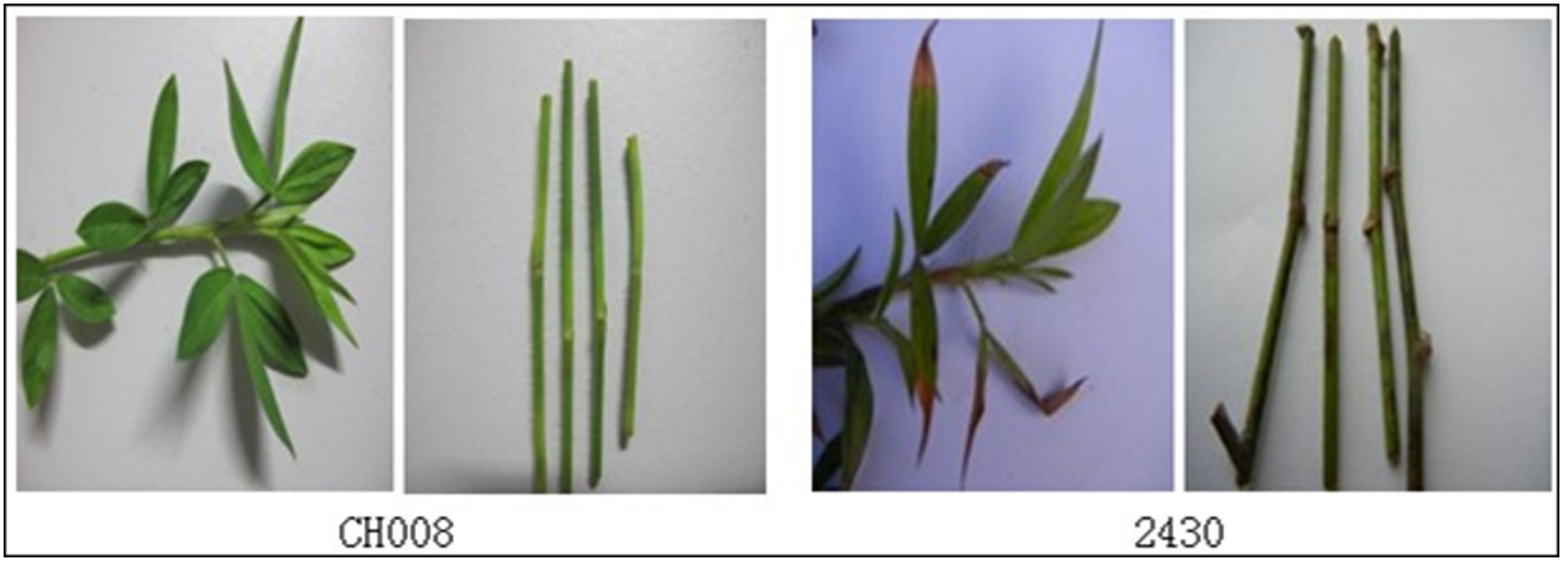
Incidence of different parts of plants after re-screening and inoculation.

For re-screening, we selected defective mutant strains whose pathogenicity was lost when preliminary screening was performed. The potted *S. guianensis* was inoculated again. Comparison of different re-screening inoculation methods showed that the most appropriate re-screening method was spraying the wild strain CH008 and transformant spore liquid to punctured parts of leaves of *S. guianensis* for verification and selection of mutants with lost pathogenicity. This method was used to re-screen five mutants with completely lost pathogenicity. The results were similar to those of preliminary screening. For example, mutant 2430 was used to infect *S. guianensis* plants. Two weeks later, pathogenic symptoms appeared. Scabs were observed on some leaves and stems of plants, and slowly expanded to most of the stem leaves (Figure 13).

#### Cloning of T-DNA insertion flanking sequences of mutant strains with pathogenic defects

**Figure 14:**
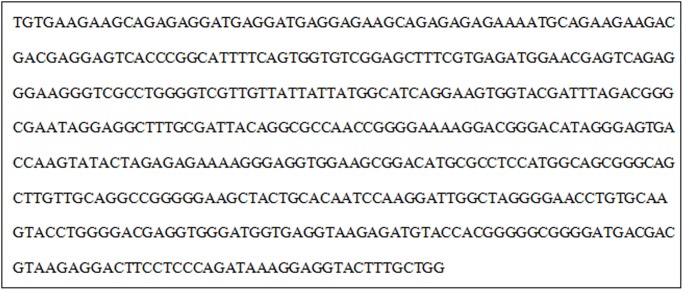
Flanking sequences of T-DNA at the insertion sites of mutant 2430.

TAIL-PCR amplification was used in 15 mutants with reduced pathogenicity and defects, and three transformants with peculiar strips were obtained, i.e., *Anthrax* transformants 2430, 913, and 3521. Only transformant 2430 was successful in transformation and sequencing; and the flanking sequences of 476 bp were obtained (Figure 14).

**Figure 15:**
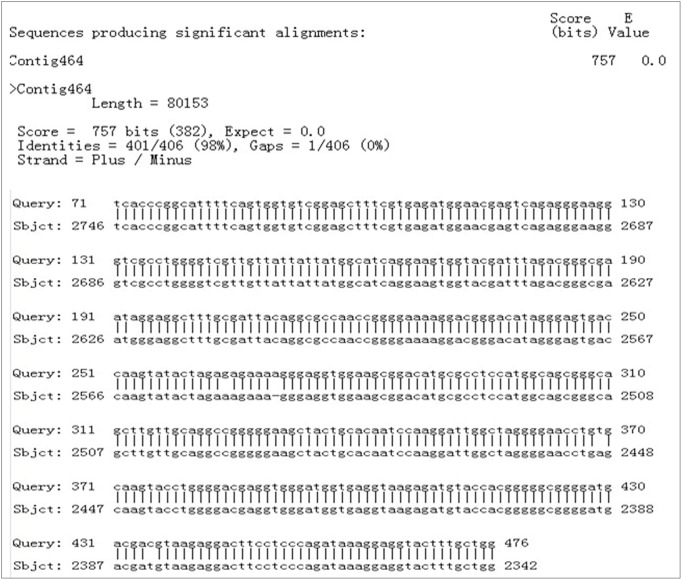
BLAST results of T-DNA flanking sequences in NCBI.

BLAST was used to compare the sequence with the sequenced *Colletotrichum* whole genome database (unpublished), and the website ‘The FGENESH Program’ (Softberry Inc., Mount Kisco, NY, USA; http://linux1.Softberry.com/berry.phtml) was used to predict its functions. Therefore, a hypothetical gene in the regional code of the nucleotide sequence was noted. BLAST was subsequently adopted to compare the sequences in NCBI; 401 nt was completely consistent with the partial sequence of Contig464 of the database (Figure 15). T-DNA was a promoter subregion of the predicted gene. The full length of the predicted gene was 1251 bp. The code of the predicted gene was 416 aa, and the amino acid homology between the predicted gene and *Magnaporthe oryzae* gene (XP_003719674.1) was 79%. This type of gene codes aspartate transaminase. This code may play an important role in the infection process of pathogeny. Insertion of exogenous sections destroyed the gene’s functions, so the mutants exhibited lost pathogenicity.

## Discussion

In 2011, genetic transformation of over 60 fungi was realized successfully by ATMT [38]. When transformation is carried out, each fungus has optimum transformation conditions. The optimum transformation and screening conditions should be explored to construct a high-quality genetic mutant library with a large quantity in a short time period. Moreover, when high-quality and efficient transformation and optimization systems are used to construct necessary mutant libraries, manpower and material resources are not only saved but the library-establishment cycle is also shortened. Such systems can also reduce difficulty in screening and cloning related pathogenic genes [39].

The cultivation, growth status, and purity of *Agrobacterium* have significant effects on transformation, and they are important for preparing *Agrobacterium* infection liquid with high purity, vigorous growth, and powerful infection capability [40]. *Agrobacterium* in the middle and late logarithmic phases is considered to be the optimum infectious bacteria. However, the time at which different species of *Agrobacterium* reach the logarithmic phase, as well as their concentrations, can differ. In the present study, after AGL-1 was cultured in MM for 36 h and cultivated in IM for 6 h, its OD_600_ value ranged from 0.6 to 1.0. By selecting *Agrobacterium* within this range and adjusting to an appropriate concentration, the obtained transformation efficiency was high.

AS is currently the most common Vir gene inducer. Numerous studies showed an optimum inductive effect at a concentration of 50–200 µmol/L. When the pH of the culture medium containing AS is 5.0–5.6, the induction of genes at the Vir region of *A. tumefaciens* peaks [41]. IM in the present study showed a final selected concentration of 100 µmol/L and pH of 5.2. This finding verifies the conclusion drawn by Holford et al., who reported optimum transformation effects at pH 5.2 [42]. When the AS concentration was 100 µmol/L, the results were significantly higher than those at other levels. However, the transformation efficiency did not improve with increasing AS concentration. Excess AS concentrations may have toxic effects on explants, and influence further improvement in the transformation efficiency [41].

The co-culture time is one of the most important factors affecting the success of the transformation of *A. tumefaciens*. The process of transformation mediated by *A. tumefaciens* takes some time, and transformants are unable to form at a very short co-culture time [35], [43]–[45]. In the present study, the number of transformants increased with increasing co-culture time. The number of transformants peaked when *S. colletotrichum* and *Agrobacterium* were co-cultured for 3–4 d, and the results were highly significant. Continuous co-culture can result in generation of false-positive clones [46], resulting in very large bacterial colonies that cannot be selected easily [47]. Campoy et al. revealed that the number of transformants peaks when the co-culture temperature is consistent with the optimum growth temperature of acceptors; reducing or increasing the co-culture temperature reduces the number of transformants [48]. The results of this study were consistent with those observed by Campoy et al.

Furthermore, selection of transformation acceptors is critical. Many forms, such as protoplast, mycelium, and conidium, can be used as acceptor materials [26]. Acceptor strains can be transformed by exogenous genes only when they stay in the phase of cell division. High requirements must be met in the preparation of protoplast, and this process is quite complicated. Materials such as mycelium and conidium can also achieve ideal transformation efficiency. Very high cell concentration of acceptor fungi can lead to excess fungal growth, so transformants cannot be selected [24]; very high *Agrobacterium* concentrations can result in serious *Agrobacterium* pollution [37].

The results of Southern blot show that the T-DNA insertion rate in transformants was 90%. The transformants, whose T-DNA insertion was at a single site, accounted for about 67%. The rate of a single copy was much higher than that of *Colletotrichum graminicola* (16%), slightly higher than that of other anthracnose species (65%) [49], [50], but lower than that obtained by Jia Peisong [51] and Wang Haiyan [52] (100%). Single-site insertion in genome is usually expected because the derived phenotype is related to changes in single sites in the genome [24], which can help in finding marker genes from the fungal genome. Some studies implied that the insertion rate of T-DNA single copy numbers has an inverse relation with the co-culture time to some extent [53]. However, the co-culture time must not be shortened too much in single-copy insertion so that the transformation efficiency does not decrease. Moreover, Examination of a large number of mutants using PCR and Southern blot is time-consuming and laborious, and generally provides only a subjective measure of infectivity. Therefore it would be worthwhile to develop the high-throughput technologies for molecular verification of *Stylosanthes* anthracnose mutants, such as Luminex or transposon sequencing, Lin Tao et al. examined 434 signature-tagged mutagenesis mutants using Luminex-based multiplex PCR, which is an efficient and timesaving method [54]–[56].

This research showed that the growth rates and sporulation quantity of some transformants changed. T-DNA insertion causes inactivation for a certain gene of transformants, and this variation results in changes in other characters. However, most transformants did not demonstrate a significant difference from untransformed strains. Conidial generation is a premise for infection and pathopoiesia of many pathogenic bacteria; the quantity and germination rate of conidia, as well as appressorium formation, affect their pathogenicity to some extent [57], [58]. In the future, relations among the pathogenicity of transformants, growth rate of mutants, sporulation quantity, conidial germination, appressorium formation, and pathopoiesia will be further examined.

The flanking sequences of T-DNA insertion sites were cloned. TAIL-PCR is commonly used to amplify unknown flanking sequences of the known T-DNA sequence. This technique can design reliable random degenerate primers that are appropriate for the background of genomes that need to be detected [59]. The present study identified the optimum random primers (i.e., AD4 and AD8), and four right flanking sequences and one left flanking sequence were obtained. The reason for these results might be the high probability at which the left border of T-DNA is cut off, which was consistent with several fungal results that have been reported [36], [49], [60]. The left border of T-DNA may be unnecessary for T-DNA transfer, whereas the right border is essential for T-DNA transfer; thus, transfer starts at the right border and continues toward the left [61].

TAIL-PCR was used in the present study to amplify the flanking sequences of five mutant genomes, and five flanking sequences were obtained. For one sequence, BLAST comparison showed that its pathogenicity was lost and the flank section amplified by t-2430 was approximately 0.5 kb. By analyzing and comparing the obtained sequences, these genes and genes of aspartate transaminase coded by the pathogenic bacterium *M. oryzae* demonstrated high homology. This enzyme mainly exists in the mitochondria of cells, and exerts important catalytic actions during nitrogen metabolism. The insertion of exogenous sections may alter the functions of genes, affect the coding of transferase, disturb some important metabolisms of the mutant, and result in the loss of pathogenicity in pathogenic infection processes.
